# Surveillance and Control of *Aedes albopictus* in the Swiss-Italian Border Region: Differences in Egg Densities between Intervention and Non-intervention Areas

**DOI:** 10.1371/journal.pntd.0004315

**Published:** 2016-01-06

**Authors:** Tobias T. Suter, Eleonora Flacio, Begoña Feijoó Fariña, Lukas Engeler, Mauro Tonolla, Lêda N. Regis, Maria A. V. de Melo Santos, Pie Müller

**Affiliations:** 1 Department of Epidemiology and Public Health, Swiss Tropical and Public Health Institute, Basel, Switzerland; 2 University of Basel, Basel, Switzerland; 3 Gruppo Cantonale di Lavoro Zanzare, Canton of Ticino, Canobbio, Switzerland; 4 Laboratory of Applied Microbiology, University of Applied Sciences and Arts of Southern Switzerland, Bellinzona, Switzerland; 5 Department of Entomology, Centro de Pesquisa Aggeu Magalhães-FIOCRUZ, Recife, Pernambuco, Brazil; Universidad de Buenos Aires, ARGENTINA

## Abstract

**Background:**

*Aedes albopictus*, the Asian tiger mosquito, originates from the tropical and subtropical regions of Southeast Asia. Over the recent decades it has been passively spread across the globe, primarily through the used tyre trade and passive transportation along major traffic routes. *A*. *albopictus* is a proven vector for many arboviruses, most notably chikungunya and dengue, with recent outbreaks also in continental Europe. In southern Switzerland, in the Canton of Ticino *A*. *albopictus* was spotted for the first time in 2003. Since then the local authorities have implemented a control programme based on larval source reduction. Despite these efforts, mosquito densities have increased over the last decade, casting doubts on the effectiveness of such larval control programmes.

**Methodology/Principal Findings:**

The Italian communities just across the Swiss-Italian border lack a control programme. This motivated us to compare the intervention and the non-intervention areas side by side in an attempt to find evidence for, or against, the effectiveness of larval *A*. *albopictus* control. Using ovitraps and a randomised sampling scheme, we examined the seasonal and spatial abundance of *A*. *albopictus* in sylvatic and urban environments across the Swiss-Italian border in 2012 and 2013. In the urban environments of the non-intervention area, egg densities were 2.26 times higher as compared to the intervention area. In the sylvatic environments, as compared to the urban environments, egg densities were 36% in the intervention area and 18% in the non-intervention area.

**Conclusions/Significance:**

Though alternative explanations are also valid, the results support the hypothesis that the Ticino intervention programme does have an impact. At the same time the data also suggest that current larval interventions fall short in gaining full control over the mosquito, calling for the evaluation of additional, or alternative, approaches. Ideally, these should also consider inclusion of the neighbouring Italian communities in the surveillance and control efforts.

## Author Summary

The Asian tiger mosquito (*Aedes albopictus*) has gained increased attention in public health because it is a globally spreading, highly invasive mosquito species that may transmit several viruses. Outside of its original range in Southeast Asia it has been increasingly implicated in local transmission of chikungunya and dengue fever in many places including La Réunion, continental Europe, the Americas and Japan. The Asian tiger mosquito lays eggs that are adapted to desiccation and colder climate. This, together with the mosquito’s ability to breed in almost any small, stagnant water body, makes its control extremely difficult, and there is much debate as to what interventions would be effective. This motivated us to compare the occurrence of the Asian tiger mosquito in southern Switzerland, where a mosquito surveillance and control programme is in place, with its neighbouring Italian districts where no such programme exists. The Swiss programme is based on public awareness campaigns to remove breeding sites and the use of insecticides against larvae. Using specialised traps that collect eggs from egg laying female mosquitoes, we found 2.26 times more *A*. *albopictus* eggs in the non-intervention area. The results support the hypothesis that targeting larval sources does have a significant impact.

## Introduction

*Aedes (Stegomyia) albopictus* (Skuse, 1894), the Asian tiger mosquito, originates from the tropical and subtropical regions of Southeast Asia. During recent decades this mosquito species has spread to North America, Europe, Latin America and Africa, primarily by the transport of dormant eggs in used tyres [1] and through the importation of *Dracaena sanderiana* plants, also known as “lucky bamboo” [2]. At the regional level the mosquito is further passively dispersed through adults displaced by vehicles along traffic routes such as motorways [3].

Under laboratory conditions, *A*. *albopictus* is a competent vector for at least 26 arboviruses, notably chikungunya, dengue, yellow and West Nile fever [4,5]. *A*. *albopictus* is also of veterinary significance because it is equally a competent vector for *Dirofilaria immitis*, a nematode that causes dirofilariosis in dogs [4]. Therefore, the establishment of *A*. *albopictus* represents a potential threat for both public and veterinary health. How realistic this threat is also for mainland Europe has been clearly demonstrated by several reports of autochthonous chikungunya and dengue cases over the recent years. In 2007, an outbreak of chikungunya associated with the establishment of *A*. *albopictus* occurred in Ravenna, Italy, with over 200 confirmed cases [6,7]. More recently, between August and September 2010, autochthonous cases of dengue have been reported from Croatia and metropolitan France with *A*. *albopictus* deemed responsible for its transmission [8,9]. In the same year, two people became also infected with the chikungunya virus in Fréjus, France [10]. Then additional autochthonous dengue cases were reported from southern France in 2013 [11] and again in 2014, alongside new cases of chikungunya [12].

In Italy, *A*. *albopictus* was first detected in Genoa in 1990 from where it spread to many parts of Italy, including the border region south of Switzerland [13]. In response to its presence in northern Italy an *A*. *albopictus* surveillance programme was put in place by the local authorities in southern Switzerland in the Canton of Ticino (in the following simply called Ticino) in 2000. Three years later, the first *A*. *albopictus* eggs were detected [14]. As increasing egg numbers were detected between 2003 and 2006, the surveillance effort was gradually intensified and control measures implemented [14]. Control measures entailed removing of potential breeding sites and use of larvi- and adulticides. In the following years the estimated *A*. *albopictus* density was still low, suggesting that individual adult mosquitoes had been sporadically introduced from Italy but had not yet established a sustained population in Ticino. Yet, in 2007 the situation changed significantly, when a dramatic increase of positive mosquito traps in Chiasso, right at the Swiss-Italian border, was observed, indicating that a local mosquito population had then been established [14,15].

In 2007 the monitoring system consisted of 292 oviposition traps (ovitraps) that were regularly controlled, covering a defined area of approx. 4.6 km^2^. Ovitraps are a widely used tool for the surveillance of container breeding *Aedes* [14,16–19] as they are sensitive, relatively inexpensive and easy to maintain [20][16,18]. The ovitrap is a device that consists of a water-filled black bucket with a piece of wood, or styrofoam, onto which female mosquitoes may deposit their eggs. In Ticino, the ovitraps used consist of a flower pot filled with water into which a wooden strip is plunged for the females to lay eggs [14]. The traps are set within communities as well as at lay-bys and service areas along the motorway E35 [21]. The E35 is a south-north European route that runs from Rome (Italy) to Amsterdam (the Netherlands). In addition, places with stagnant water that cannot be averted otherwise were treated with *Bacillus thuringiensis* var. *israelensis (Bti)*, a biological control agent for larval mosquito stages [22].

During the last years, the ovitrap network has been continuously expanded and adapted. Today, over 1,000 ovitraps are deployed within the frame of the Ticino surveillance and control programme covering an area of approx. 60 km^2^. The traps are inspected biweekly and the number of positive traps serves as an indicator if and where the application of insecticide would be necessary [14]. In addition, information campaigns are carried out to raise public awareness in order to sensitise residents for the occurrence of *A*. *albopictus* and to eliminate potential breeding sites from their private properties. Despite these measures *A*. *albopictus* densities have still increased in Ticino over the last decade [14].

Larval source reduction by removing water containers that may serve as breeding sites is considered the best method for the control of *A*. *albopictus* by several authors [23,24]. Studies from North Carolina [25], Spain [26] and New Jersey [27] reported that source reduction campaigns resulted in a temporary suppression of immature *A*. *albopictus*. Indeed, Bartlett-Healy et al. [28] showed that artificial containers on private properties are the most productive sources for the emergence of *A*. *albopictus*, highlighting the importance of public involvement in the overall control effort. Awareness campaigns showing the public how to identify and eliminate potential breeding sites from their properties have become an integral component of *Aedes* mosquito control [20]. Such campaigns go often hand in hand with larvicide treatments and spraying of insecticides targeting adult mosquitoes. Comparing different intervention approaches, Fonseca et al. [27] concluded that careful source reduction by trained personnel, in combination with efforts to educate the public in removing breeding sites, results in a significant decrease in adult *A*. *albopictus* numbers.

Despite the above evidence there is still much debate as to how effective such larval control measures really are, particularly in areas where mosquitoes are continuously re-introduced such as being the case in southern Switzerland. This motivated us to examine the potential impact of the current surveillance and control programme by comparing relative mosquito densities between Ticino and two neighbouring Italian provinces where ecological parameters are comparable; yet, no intervention programme is in place.

## Methods

### Study area

**Fig 1:**
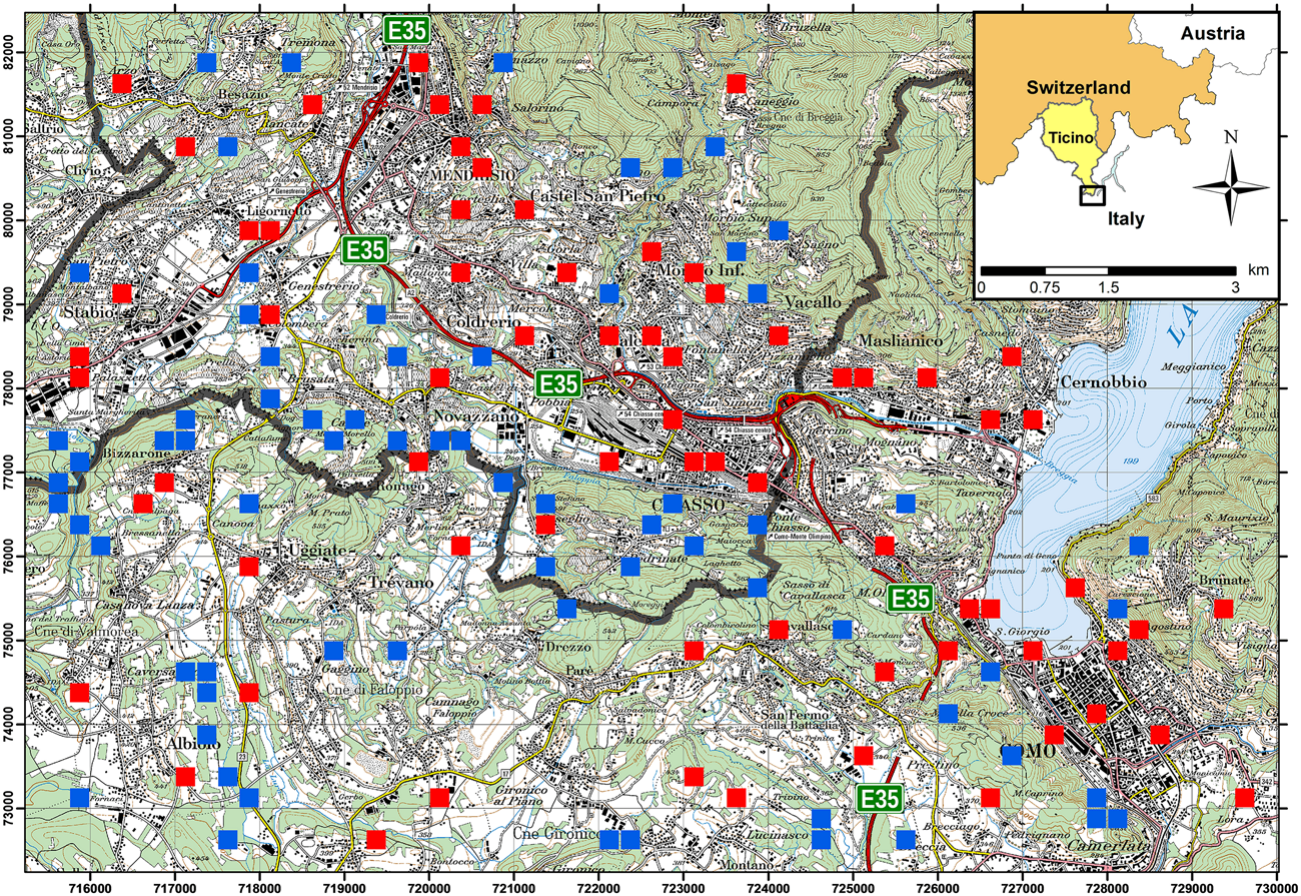
Study area and ovitrap positions. The red and blue squares represent sampling grid cells in urban (red) and sylvatic (blue) environments. In each country 35 grid cells were randomly allocated to either the urban or sylvatic environment. Within selected grid cells two ovitraps were placed at a minimum distance of 50 m between them to avoid interference in mosquito attraction. In total, there were 280 ovitraps (2 countries x 2 environments x 35 cells x 2 ovitraps). The thick grey line denotes the Swiss-Italian border with the intervention area (Ticino, Switzerland) in the North and the non-intervention area (Lombardy, Italy) in the South. The orange line, crossing the Swiss-Italian border, shows the European route E35. The numbers at the left and at the bottom indicate the Swiss km co-ordinates.

Field surveys were carried out from July to November 2012 and from May to November 2013. The study area enclosed the southernmost border region of Ticino, the Mendrisiotto district, and the provinces of Varese and Como in Lombardy, Italy (Fig 1). Hereafter, the part of the study area in Ticino is called the “intervention” area and that of Varese and Como the “non-intervention” area. In total, the study area covered a surface area of 118 km^2^; 65 km^2^ on the Italian side and 53 km^2^ on the Swiss side of the border. The difference in the surface areas were to make up for places that were either inaccessible or covered by the Lake of Como.

The landscape of the study area is similar on both sides of the border and dominated by deciduous forests and agriculture. Approximately 20% are covered by buildings or roads. Population densities are almost equal and are 440 and 480 inhabitants per km^2^ in the Ticino and the Lombardy part, respectively [29,30].

The traffic-intense European route E35 runs through the study area, connecting the South of the continent with North-western Europe. On average, on a single work day over 62,000 people cross the Swiss-Italian border, mostly by car [31,32]. Most of the people crossing the border commute to Switzerland for work.

The climate in the study area is continental with relatively mild temperatures, yet distinct annual seasons. Mean annual temperature and rainfall are 11.1°C and 1,311 mm [33]. Besides its relatively sunny weather, the region is also well known for its heavy thunderstorms during the summer.

Using the ArcGIS version 10.0 (ESRI Inc., USA) geographic information system (GIS) software a grid with 250 m by 250 m cells was virtually superimposed over the study area. From this grid, all grid cells within a lake and those that were inaccessible in the field were excluded from sampling. The remaining grid cells were then stratified into “urban” and “sylvatic” environments. A cell was classified as sylvatic if at least 50% of the surface were covered with trees, and vice versa. For each of the four combinations of area and environment 35 cells were randomly picked from the grid to avoid sampling bias. For this purpose the cells were first numbered through and then the numbers drawn using a random number generator. The total number of cells included in the study was chosen on the basis of a power calculation that used simulation methods described in Johnson et al. [34]. For this exercise we assumed a minimal effect size of 10% difference in egg counts between the two countries and a power of 1-β = 0.8.

### Ovitrap sampling and species identification

Relative densities of *A*. *albopictus* were estimated using ovitraps. The traps mimic breeding sites, attracting gravid females to deposit their eggs. In the present study, an ovitrap consisted of a 1.5 l, black plastic flower pot, filled with 1.2 l tap water. Three small holes with a diameter of 5 mm were drilled at equal distances, 2 cm below the rim, to prevent the trap from being flooded by rain. A wooden strip made of untreated beech wood was placed inside the pot so that it was partially submerged and partially sticking out of the water. The strip measured 20 cm x 2.5 cm x 0.5 cm. In order to prevent the ovitraps from becoming potential breeding sites larvicide granules of *Bti* (VectoBac, Valent BioSciences, USA) were added. The strips, water and *Bti* were replaced biweekly. When replaced, the traps were cleaned and the wooden strips wrapped in clingfilm for transportation and preservation. Each strip was labelled with the date and a unique code together with additional information related to the trap condition and the presence of larvae. The final trap position within the assigned sampling grid cell was chosen in the field. Traps were placed at shaded, wind protected locations that, in the optimal case, were surrounded by green vegetation as done in previous studies (e.g. [14,27]). All traps were geo-referenced with a handheld GPS device (nüvi 1390, Garmin, Switzerland).

In the laboratory, the strips were inspected for the presence of mosquito eggs using a stereo microscope (EZ4D, Leica Microsystems, Germany) and, where present, the number of eggs counted. During the first season in 2012, eggs were identified to species level by morphology. At that time only two container-breeding mosquito species, *A*. *albopictus* and *A*. *geniculatus*, were known to occur in the region. Both species can easily be distinguished by morphology [20,35]. As a quality control measure an additional identification method was introduced for the 2013 mosquito season. Here, for each collection round, eggs from two randomly selected positive traps were also analysed by matrix-assisted laser desorption/ionization mass-spectrometry (MALDI TOF MS) [35]. Only eggs were chosen for the analysis that had previously been morphologically determined as being *A*. *albopictus* and, where present, were still intact. For MALDI-TOF MS three to five apparently intact eggs were carefully removed using forceps from the ovitrap strips and then transferred to 1.5 ml Eppendorf tubes. The samples were sent to Mabritec SA (Riehen, Switzerland) where they were prepared and analysed according to the protocol described in Schaffner et al. [35].

### Data analysis

The numbers of *A*. *albopictus* eggs on each wooden strip were counted and recorded in an Excel data base together with additional information such as the trap location, date, condition of the trap, etc. Data were then imported into the GIS software ArcGIS Version 10.1 (ESRI Inc., USA) to produce spatio-temporal density maps. For statistical analysis, data were loaded into the freely available software R, version 3.1.2 [36].

**Fig 2:**
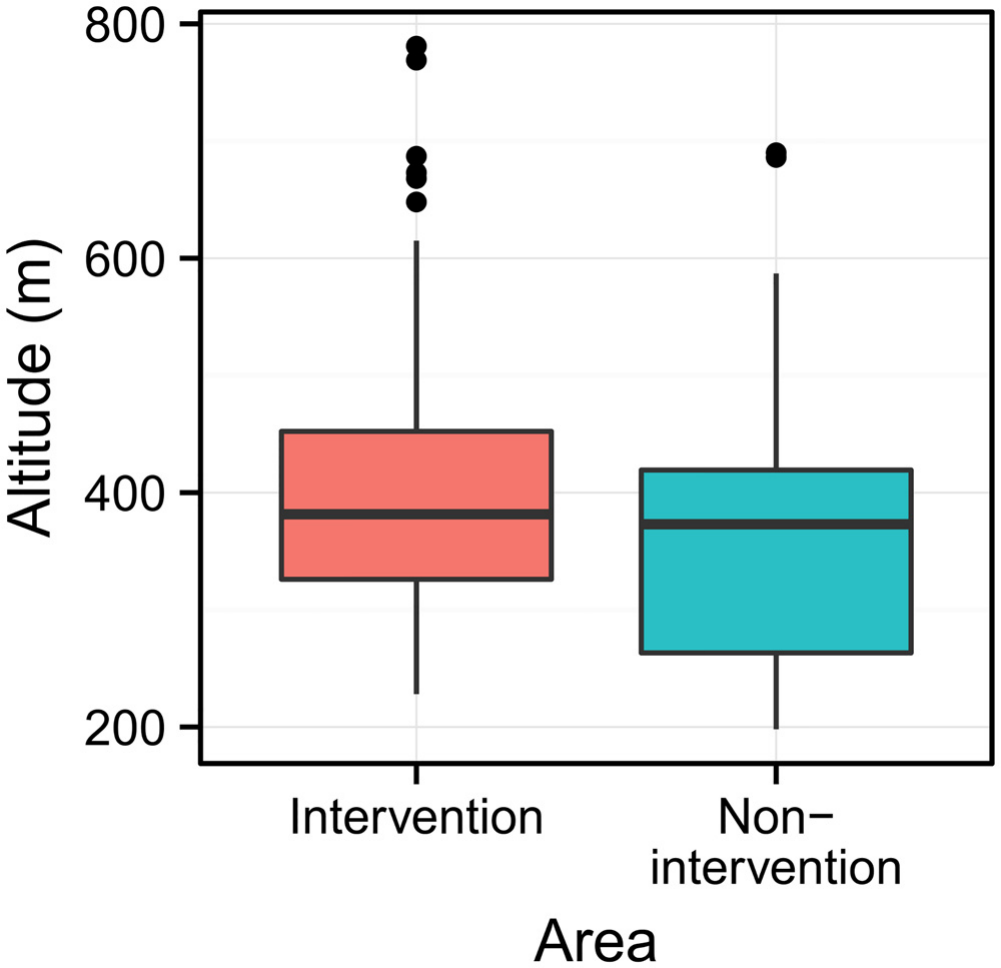
Altitude range of trap positions. The boxplots show the distribution of the altitude above sea level for the 140 ovitraps in each of the two areas. The boxes represent the interquartile distances (IQD), while the centrelines through each box show the medians. The dots indicate outliers and the whiskers extend to the extreme values of the data, calculated as ±1.5 x IQD from the median.

Relative egg densities per trap were modelled by a zero-inflated negative binomial (ZINB) regression model using the R package “glmmADMB” [37,38]. The ZINB accounted for an excessive number of zeros in the ovitrap count data. In the ZINB model, the outcome was the biweekly egg count per trap, while the predictors “area” (non-intervention vs. intervention) and “environment” (urban vs. sylvatic) and their interaction were included as fixed effect terms. To account for the slight bias in altitude towards higher elevations in the intervention area (Fig 2) and the potential relationship between altitude and temperature, a predictive term for “altitude” was also included in the model. Altitude was entered as metres above sea level. As egg counts were repeatedly (i.e. biweekly) measured for the same ovitrap, an intercept was included for “trap” as a random term in the ZINB model, accounting for correlations in the number of eggs caught in the same trap. Also included as a random term was an intercept for the week in which the traps were replaced in order to account for seasonal variations. The model was also inspected for signs of spatial correlations in the residuals using the variogram function in the R package “gstat”, version 1.0–19 [39]. The statistical graphics were produced with ggplot2, version 1.0.0 [40]. The level of significance was set at α = 0.05.

## Results

**Fig 3:**
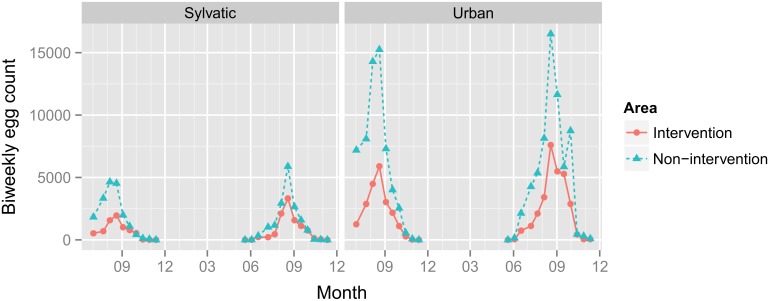
Temporal distribution of *Aedes albopictus* in the Swiss-Italian border region. The numbers of *A*. *albopictus* eggs found in the ovitraps are shown as sums over all 70 traps for each combination of environment and area. In the calendar week 38 in 2013, an unusually high number of ovitraps was dysfunctional (e.g. traps were found turned over, damaged or missing; S1 Table), explaining the sudden drop in the curve for the non-intervention area in the urban environment.

In 2012, ovitrap collections ran over 20 weeks (i.e. 10 rounds) from July to November, while the survey covered 26 weeks (i.e. 13 rounds) from May to November in 2013. The first eggs in the season were found in early June, followed by a steady increase with a peak between 19 and 26 August. In September, egg counts dropped again and eventually ceased in mid-November (Fig 3).

From the potentially 6,440 available strips for the analysis (280 traps x 23 rounds), 357 (5.5%) have gone missing (Table 1 and S1 Table); either they have been taken from the traps or the traps themselves became dysfunctional (e.g. traps were found turned over or missing completely). From the remaining 6,083 strips, 2,508 (41.5%) were positive for *A*. *albopictus*, 689 (11.4%) for *A*. *geniculatus* and 333 (5.5%) for both species. While for *A*. *albopictus* a total of 224,728 eggs were counted, egg numbers were not recorded for *A*. *geniculatus*, only whether eggs were present or absent.

**Table 1:** Summary of the biweekly *Aedes albopictus* egg counts.

Area	Year	Strips analysed	Strips missing	Positive strips	Egg count per strip					
					Minimum	1^st^ quartile	Median	Mean	3^rd^ quartile	Maximum
Intervention	2012	1,370	30	563 (44.1%)	0	0	0	20.6	16	441
	2013	1,677	143	550 (32.8%)	0	0	0	23.3	13	1,333
Non-intervention	2012	1,375	25	707 (51.4%)	0	0	1	56.2	51.5	1,537
	2013	1,661	159	688 (41.4%)	0	0	0	48.2	41	1,039

In 2012, egg counts per trap ranged from 0 to 1,537 in the non-intervention area (i.e. Lombardy, Italy) and from 0 to 441 in the intervention area (i.e. Ticino, Switzerland). In 2013, egg counts ranged in the non-intervention and intervention area from 0 to 1,039 and from 0 to 1,333, respectively. Egg counts were generally higher in the non-intervention area (Table 1). In all (i.e. 20) instances the morphological identification was confirmed by MALDI-TOF MS.

Remarkably, *A*. *albopictus* eggs were found across the whole altitude range (Fig 2) and were even repeatedly found at higher altitudes up to 781 m above sea level (S1 Table).

**Fig 4:**
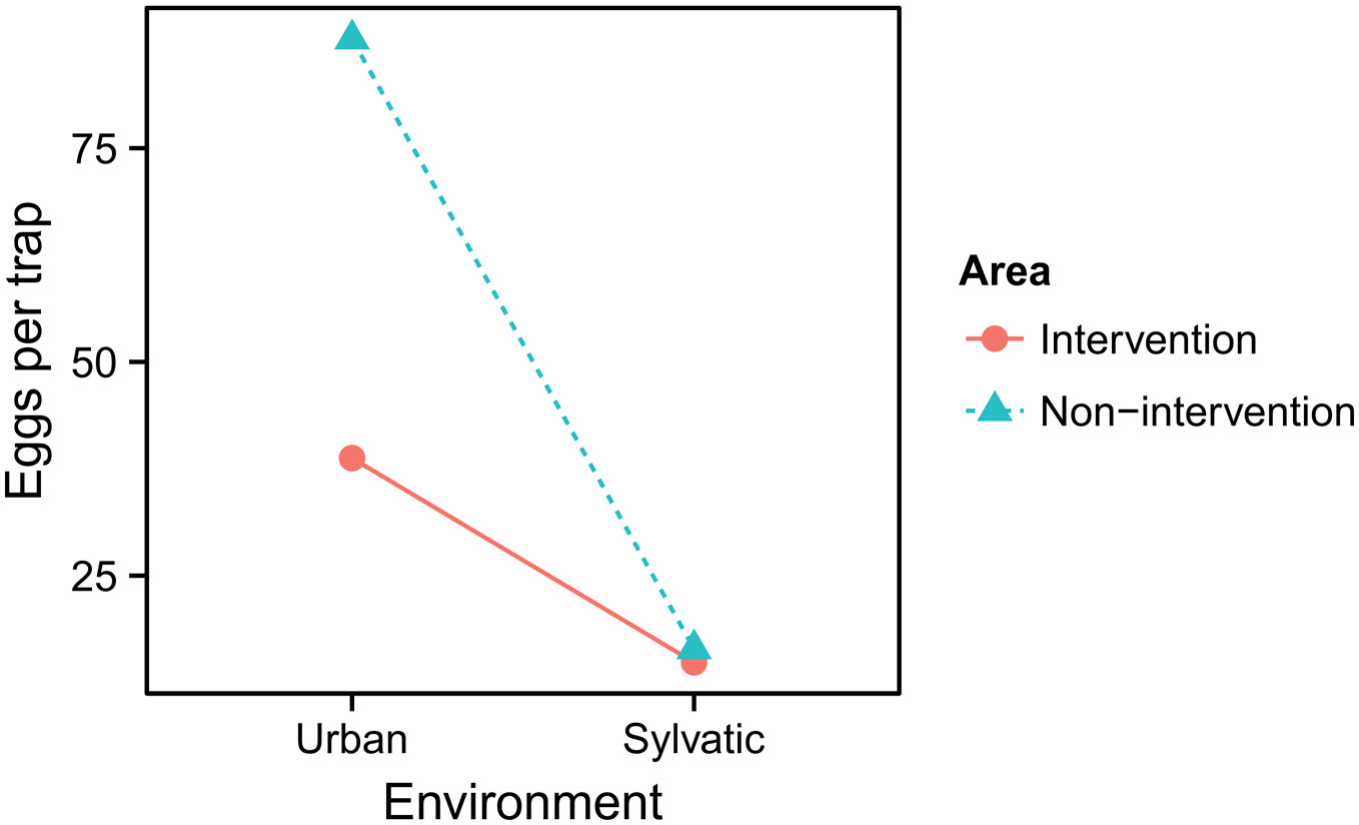
Effects of “area” and “environment” on average egg counts. The difference in average egg counts between the urban and sylvatic environments in the intervention area was half the difference between the environments in the non-intervention area. Note that the average egg numbers represent the mode from the back-transformed coefficients.

In the urban environment, the average ratio in egg densities between the non-intervention and the intervention area was 2.26 (95% confidence interval, CI: 1.40–3.65; Fig 4 and Table 2). Mosquito eggs were also detected in the sylvatic environment, although, as compared to the urban environment, the counts were much lower. The average ratios between the sylvatic and the urban environments were 0.36 and 0.18 in the intervention and in the non-intervention area, respectively. In the model, the difference in these ratios is accounted for by the interaction term (Table 2) with an estimated ratio of 0.504 (CI: 0.254–0.997) and graphically illustrated in Fig 4. In addition, the model improved by adding a term for altitude; an increase of altitude by one meter decreases egg counts by a ratio of 0.995, that is by 0.54% (95% CI: 0.37%– 0.71%). The model did, however, not improve when adding “year” as a term, indicating that egg counts did not significantly differ between the two years (χ^2^ = -2.6, *p* = 1). Moreover, inspecting the residuals for spatial correlations did not detect violation of independence.

**Table 2:** Result summary for the zero-inflated negative binomial model (ZINB). The ZINB predicts the average number of eggs caught in an ovitrap as a function of the predictors.

Predictor	Coefficient β (log_2_)	SE(β) (log_2_)	z-value	p-value
Intercept	3.675	0.729	5.04	< 0.0001
Area (non-intervention)	0.817	0.244	3.35	< 0.001
Environment (sylvatic)	-1.021	0.253	-4.04	< 0.0001
Interaction: Area (non-intervention) x Environment (sylvatic)	-0.686	0.348	-1.97	< 0.05
Altitude	-0.005	0.001	-6.13	< 0.0001

Negative binomial dispersion parameter: 0.651 (SE = 0.035). Zero-inflation: 0.315 (SE = 0.014). The variances of the random intercepts for “trap” and “week” were 1.904 (SD = 1.38) and 9.046 (SD = 3.008), respectively. Number of observations: total = 6,083; trap = 280; week = 23.

**Fig 5:**
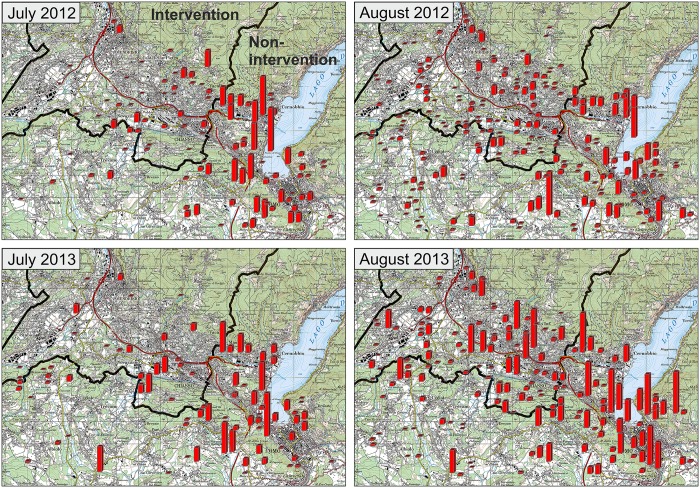
2012 and 2013 early and peak season trapping data. The size of the red bars represents the number of eggs found in the ovitrap (the smallest bars represent 1 to 50 eggs, the largest 900–1,500). The thick black line marks the Swiss-Italian border; the red line represents the European motorway E35. To enable visibility of all bars, some are slightly shifted to the right.

When plotting the positive traps on the geographic map, it becomes apparent that not only the numbers of eggs were higher in the non-intervention area but, equally, more traps were positive (Fig 5). The picture remained the same in both years and in the early (July) and late (September) mosquito season. Combining egg counts from both seasons, 32.4% (72,869 eggs) of the *A*. *albopictus* eggs were collected alone in the city of Como. In Switzerland the communities of Chiasso and Balerna, which are located at (i.e. Chiasso) or very close to (i.e. Balerna) the border, had the highest *A*. *albopictus* egg counts. Over both sampling periods, total egg numbers in Chiasso and Balerna were 12,637 and 12,212, respectively. Together they represent 11% of the total *A*. *albopictus* egg count. As traps were, however, distributed randomly to make inference about the whole region these numbers have to be interpreted with caution.

## Discussion

Our results show that in the urban environment the *A*. *albopictus* egg density was 2.26 times higher in the non-intervention area, on the Italian side of the border, as compared to the intervention area in Ticino. We also found that the ratio in egg densities between the urban and sylvatic environment was twice as high in the non-intervention area. Together, although not yet fully conclusive, the results are in line with the hypothesis that the Ticino control strategy of larval source reduction does affect *A*. *albopictus* in the urban environment.

In Ticino, the backbone of the *A*. *albopictus* control programme consists of larval source reduction through public awareness campaigns and larviciding [14]. Public awareness campaigns use multiple communication channels, including the media, internet and leaflets. As a result, artificial containers such as flower pots or water storage tanks are routinely turned over or covered. Larviciding consists of monthly applications of diflubenzuron or weekly treatments with *Bti* in the public space during the main mosquito season from May to October. Citizens are also encouraged to treat water bodies in their gardens that may not be avoided otherwise with commercially available *Bti* pellets. Certain areas such as school yards, or areas from where imported cases of chikungunya or dengue have been reported, are also sprayed with permethrin targeting adult mosquitoes [14]. In contrast to the coordinated efforts in Ticino, we are not aware of such a control programme in the Italian communities close to the Swiss-Italian border. We, therefore, hypothesise the observed differences in egg densities being attributable to the bias in mosquito control efforts. Preliminary results (S1 File) as well as the personal experience from the field made by the authors do suggest more breeding sites being present in the Italian communities. It would have been desirable to systematically quantify the presence and characteristics of breeding sites, and include in the analysis the actual amount of insecticides applied in both the intervention and non-intervention areas. Unfortunately, our resources were limited; and hence including such data was beyond the scope of the current study. However, it has to be noted that even by having that data available we would still not be able to reach a conclusive answer as the observations might still be correlated to yet another unknown variable. A much more powerful approach would be a trial in which the impact is measured in response to the implemented intervention.

Despite the above limitations, the results are in line with the few previous studies that have investigated the effects of larvi- and adulticiding [23–27]. It is also recognised that the positive effect of interventions in public areas may be strongly boosted by involving the general public in removing potential breeding sites from their own properties. Correspondingly, Vanlerberghe et al. [41] found that by engaging the public in reducing larval breeding sites in a routine vector control programme can reduce *Aedes* infestations by 50–75%. The other positive effect is this concept ensures better embedding of mosquito control in the social, cultural, political and economic context [42].

In the present study we used egg counts from ovitraps to estimate and compare *A*. *albopictus* densities because these traps are sensitive at low mosquito densities [43], are cheap and run independently of electricity or a source of carbon dioxide. There are, however, concerns over the validity of using ovitraps for density estimates because a single female may place its eggs in multiple sites [37], or the ovitraps may compete with nearby breeding sites (see e.g. [44]). Intriguingly, Carrieri et al. [45] found that ovitrap data were a reliable alternative for the mean number of biting females per unit area as well as larval productivity. Similarly, Facchinelli et al. [46] found a good correlation between sticky trap catches of adults and egg counts in ovitraps. Perhaps some studies might have failed in finding a relationship between egg counts and other sampling methods due to the use of derived statistics from non-normally distributed egg counts rather than working directly from the actual counts as done here.

In the present study, ovitraps even up to 781 m altitude were found repeatedly positive for *A*. *albopictus* eggs throughout the entire season. It has previously been assumed that eggs are unlikely to survive winter conditions at such altitudes even in warmer climatic conditions [47]. Although we cannot fully exclude rapid re-colonisation in spring or repeated re-introductions during summer, our observations suggest local reproduction rather than sporadic introductions. Altitude was also included as a covariate in the statistical model to account for the heterogeneity in elevation, and to some extent also temperature, across the entire study area.

In its native range *A*. *albopictus* is a tree hole-breeding mosquito, yet it is perfectly adapted to the man-made urban environment [48], where blood sources and (artificial) breeding sites are more readily available, demonstrated here by the much higher mosquito densities in the urban environments. As a consequence, focusing control efforts in urban areas is expected to be more effective though forests may still serve as reservoirs. Implementing control measures in forested areas is, however, even more challenging if not impossible due to the ban of using insecticides in forests [49].

Intriguingly, most ovitraps in Switzerland were still negative earlier in the season, when in Italy many traps had already been positive for *A*. *albopictus*. How can we explain this pattern? One explanation would be that the early season intervention in Ticino successfully eliminates the first mosquito generation in the year, resulting in lower reproduction. Also treatments that have been done by the end of the previous season could contribute to the observed pattern. A third explanation would be that we observe a boundary effect due to e.g. climatic constraints [50]. In the latter scenario, mosquitoes are annually re-introduced from Italy, rather than being stable overwintering populations, so that in Ticino numbers manage to pick up only later in the season. This raises the question as to what extent the Ticino *A*. *albopictus* population has firmly established in Switzerland. In other words, how many egg batches from the previous year have actually survived the winter? A study on the population genetic structure might shed light on the above question. Besides this being a question of academic interest, knowing how mosquitoes propagate and leak into the control area would also help in improving intervention strategies. In this context, Talbalaghi [51] found in the Italian region Piedmont that, without concerted actions between neighbouring municipalities, the long term effect of the control efforts were undermined. Therefore, we would strongly advocate the development and implementation of a transnational action plan for the surveillance and control of *A*. *albopictus* in the Swiss-Italian border region. Given how local residents mostly welcomed us to set the traps on their private properties and their keen interest in our work, we are very positive that an interregional action plan would receive a lot of support from the public.

### Conclusions

We found that *A*. *albopictus* egg densities in the non-intervention area on the Italian side of the Swiss-Italian border were more than twice compared to the intervention area in Ticino. Though other factors might explain the difference in mosquito densities, the present data support the hypothesis that the currently implemented surveillance and control programme in Ticino has a positive impact. Presumably public awareness is a major component in reducing *A*. *albopictus* densities. However, it remains to be shown experimentally how big the actual impact of the current interventions really is.

## Supporting Information

S1 Table Original data set with egg counts for each wooden strip. Each line corresponds to a single strip / observation. TRAP.ID = unique identifier for each trap location; WGS84.LAT and WGS84.LNG = geographical coordinates (i.e. latitude and longitude) in the World Geodetic System format WGS84; ALTITUDE = metres above sea level; AREA = area, intervention (Ticino, Switzerland) and non-intervention (Lombardy, Italy); MUNICIPALITY = municipality, the administrative division; ENVIRONMENT = “sylvatic” or “urban” environment; DATE = day when strip was removed from the trap in the field; N.ALBOPICTUS = number of *Aedes albopictus* eggs on the strip (“NA” means the strip was missing); GENICULATUS = logic variable indicating the presence of *Aedes giniculatus* eggs on the strip.
(XLSX)

S1 File Characterisation of potential breeding sites from 8 randomly selected sampling grid cells. (DOCX)
