# Supplementary material for: Surveillance and Control of Aedes albopictus in the Swiss-Italian Border Region: Differences in Egg Densities between Intervention and Non-intervention Areas
Source: PLoS Negl Trop Dis. 2016 Jan 6;10(1):e0004315. doi: 10.1371/journal.pntd.0004315 (PMC4703296; doi:10.1371/journal.pntd.0004315)
Supplement: S1 File — (DOCX) [file pntd.0004315.s002.docx]

**Supplementary File S2:** Breeding sites characterisation

In 2012, a total of eight randomly selected sampling cells have been systematically screened for *Aedes albopictus* breeding sites. The cells were located both in urban and sylvatic environments in the intervention and non-intervention areas.

Table 1: Overview of sampling cells selected for the characterisation of breeding sites.

| **Sampling cell ID^1^** | **Area** | **Community/country** | **Environment** | **Date** | **No. BS^2^** |
| --- | --- | --- | --- | --- | --- |
| 1313 | Non-intervention | Bizzarone/Italy | Sylvatic | 21.11.2012 | 10 |
| 1583 | Non-intervention | Como/Italy | Urban | 21.11.2012 | 16 |
| 2160 | Non-intervention | Como/Italy | Sylvatic | 21.11.2012 | 54 |
| 2085 | Non-intervention | Lucino/Italy | Urban | 22.11.2012 | 56 |
| 654 | Intervention | Balerna/Switzerland | Sylvatic | 21.11.2012 | 19 |
| 885 | Intervention | Balerna/Switzerland | Urban | 21.11.2012 | 50 |
| 1345 | Intervention | Chiasso/Switzerland | Sylvatic | 22.11.2012 | 17 |
| 601 | Intervention | Morbio Inferiore/Switzerland | Urban | 22.11.2012 | 10 |

^1^ The sampling cell ID refers to the grid system used for the ovitrap monitoring.

^2^ Number of identified (potential) breeding sites.

**I.) 1313: Bizzarone, Italy (sylvatic)**

Habitat description: sampling cell is located on the flank of a hill, cut by a little stream and a hiking trail. The whole area is a densely wooded (mixed forest). A road crosses the edge of the sampling cell.

Identified (potential) breeding sites: we identified two tree holes that could potentially serve as breeding sites. Alongside the road we found some rubbish in the forest and under the bushes: 3 glass bottles and 7 plastic cups. We also counted three rainwater catchments on the road.

**II.) 1583: Como, Italy (urban)**

Habitat description: a park bordering the Lake of Como. In the centre of the grid cell was the Villa Olmo, a large old building. A public swimming pool and two tennis courts were bordering the park. Wide lawns are dominating the park interspersed with trees. There are several little fountains (moving water) and sculptures.

Identified (potential) breeding sites: 4 tree holes, 8 cut lamp poles, tool shed with a leaky roof counting numerous empty flowerpots and watering cans (roof was not waterproof), 7 empty flowerpots and 36 open rain water catchments.

**III.) 2160: Como, Italy (sylvatic)**

Habitat description: scarp above the city of Como, densely wooded mixed forest, cut by a road. There was a residential building and an abandoned restaurant building surrounded by a big garden and a paddock in the centre of the sampling cell. Uphill of the grid cell was a natural protected forest area and downhill were (already outside of the sampling cell) additional residential buildings.

Identified (potential) breeding sites: generally we found a lot of waste in the bushes alongside the road, including 3 Tupperware, 13 plastic cups, 9 bottles, 8 tins, and 4 used tyres. In the big garden of the abandoned restaurant we found 14 empty flowerpots and 3 open rain barrels.

Alongside the road we found three big manholes filled with water and colonised by mosquito larvae.

We could not identify any natural breeding sites like tree holes or rock pools.

**IV.) 2085: Lucino, Italy (urban)**

Habitat description: in the centre of the sampling grid was a big old farmhouse, surrounded by a big garden; forest, horse meadows, stables and agriculture land surround the area.

Identified (potential) breeding sites: a collection point for waste was situated close to the house; there we found 6 plastic containers and 2 plastic tons. In the garden we counted 7 empty flowerpots and an open rain barrel. We counted 6 open rainwater catchments alongside the road.

**V.) 654: Balerna, Switzerland (sylvatic)**

Habitat description: the sampling grid cell was located in a natural protected area. There was a little canyon and a stream. The area is located in a valley, surrounded by wooded hills. In central position a lawn and a little street with limited traffic dominate the area. In the upper part of the sampling grid is a little fenced garden.

Identified (potential) breeding sites: 14 open rainwater catchments, 1 old bathtub in the garden, 2 tree holes and 2 empty flowerpots.

**VI.) 1345: Chiasso, Switzerland (sylvatic)**

Description: the sampling cell was a steep slope. A road wound uphill in several curves. There were few residential buildings in the lower part of the sampling cell. Above the road the area was dominated by dense bushes, called “Macchia” in Italian.

Types of breeding sites: 11 open rainwater catchments, 2 plastic cups alongside the road, 1 water-filled manhole and 3 empty flowerpots.

**VII.) 885: Balerna, Switzerland (urban)**

Habitat description: neighbourhood with residential buildings. There was a little park with a lawn, a playground and a pond.

Identified (potential) breeding sites: 42 Open rainwater catchments, 3 tree holes, 4 empty flowerpots, all the other flowerpots in the gardens were turned-over or covered.

**VIII.) 601: Morbio Inferiore, Switzerland (urban)**

Habitat description: In the centre of the sampling grid cell was a big apartment building, surrounded by a little vineyard, a sheep meadow and several trees.

Identified (potential) breeding sites: 5 open rainwater catchments, 2 drinking vessels for the sheep, 1 rain barrel, and 2 open plastic buckets.
